# A nationwide population-based cohort study to identify the correlation between heart failure and the subsequent risk of herpes zoster

**DOI:** 10.1186/s12879-015-0747-9

**Published:** 2015-01-16

**Authors:** Ping-Hsun Wu, Yi-Ting Lin, Chun-Yi Lin, Ming-Yii Huang, Wei-Chiao Chang, Wei-Pin Chang

**Affiliations:** Division of Nephrology, Department of Internal Medicine, Kaohsiung Medical University Hospital, Kaohsiung, Taiwan; Department of Family Medicine, Kaohsiung Medical University Hospital, Kaohsiung, Taiwan; Department of Radiation Oncology, Cancer Center, Kaohsiung Medical University Hospital, Kaohsiung, Taiwan; Graduate Institute of Medicine, College of Medicine, Kaohsiung Medical University, Kaohsiung, Taiwan; Department of Clinical Pharmacy, School of Pharmacy, Taipei Medical University, Taipei, Taiwan; Department of Healthcare Management, Yuanpei University of Medical Technology, Hsinchu, Taiwan; Master Program for Clinical Pharmacogenomics and Pharmacoproteomics, School of Pharmacy, Taipei Medical University, Taipei, Taiwan

**Keywords:** Heart failure, Herpes zoster, Population-based study, Taiwan National Health Insurance Research Database

## Abstract

**Background:**

The association between heart failure (HF) and herpes zoster has rarely been studied. We investigated the hypothesis that HF may increase the risk of herpes zoster in Taiwan using a nationwide Taiwanese population-based claims database.

**Method:**

Our study cohort consisted of patients who received a diagnosis of HF in 2001 ~ 2009 (*N* = 4785). For a comparison cohort, three age- and gender-matched control patients for every patient in the study cohort were selected using random sampling (*N* = 14,355). All subjects were tracked for 1 year from the date of cohort entry to identify whether or not they had developed herpes zoster. Cox proportional-hazard regressions were performed to evaluate 1-year herpes zoster-free survival rates.

**Results:**

The main finding of this study was that patients with HF seemed to be at an increased risk of developing herpes zoster. Of the total patients, 211 patients developed herpes zoster during the 1-year follow-up period, among whom 83 were HF patients and 128 were in the comparison cohort. The adjusted hazard ratio (AHR) of herpes zoster in patients with HF was higher (AHR: 2.07; 95% confidence interval (CI): 1.54 ~ 2.78; *p* < 0.001) than that of the controls during the 1-year follow-up. Our study also investigated whether HF is a gender-dependent risk factor for herpes zoster. We found that male patients with HF had an increased risk of developing herpes zoster (AHR: 2.30 95% CI: 1.51 ~ 3.50; *p* < 0.001).

**Conclusions:**

The findings of our population-based study suggest that patients with HF may have an increased risk of herpes zoster. These health associations should be taken into consideration, and further studies should focused on the cost-effectiveness of the herpes zoster vaccine should be designed for HF patients.

## Background

Herpes zoster manifests as characteristic painful vesicular skin lesions and related neurological disorders usually unilaterally grouped and limited to 1 to 3 dermatome. Herpes zoster is caused by spontaneous reactivation of a latent varicella-zoster virus (VZV) that resides in sensory ganglia and dorsal nerve roots following a varicella infection [1]. Although most herpes zoster attacks resolve spontaneously, several neurologic complications can develop, including post-herpetic neuralgia, cranial nerve palsies, myelitis, encephalitis, ventriculitis, and vasculopathy [2]. Post-herpetic neuralgia, the most common complication, results in functional disability and a reduced quality of life. Herpes zoster also increases the risk of stroke and cancer according to population-based studies [3-6]. Therefore, herpes zoster has important impacts on the health of adults, particularly the elderly, and on health systems.

The estimated lifetime risk of herpes zoster is 10% ~ 30%, and the incidence and severity markedly increase after the age of 50 years [7-11]. The incidence of herpes zoster in the general population is 1.2 ~ 4.9 cases per 1000 person-years [11-13] and the post-herpetic neuralgia incidence is 0.42 per 1000 person-years [1]. Old age is the most well-known risk factor for herpes reactivation [14]. Other established risk factors for herpes zoster include diabetes mellitus, chronic obstructive pulmonary disease (COPD), malignancy, and immune-compromised conditions (eg., patients with AIDS, systemic lupus nephritis, and rheumatoid arthritis, and transplant recipients,) [15-25]. Although the mechanism of reactivation of latent VZV remains unclear, decreasing cellular immunity to VZV predisposes one to the recurrence of herpes zoster [26,27].

Heart failure (HF) has high prevalence rate among elderly patients and increases with aging [28,29]. Disseminated zoster in elderly patients with hypertension and HF was previously reported [30]. Furthermore, higher herpes zoster risk among patients with cardiovascular disease was also found [31]. There are no published data regarding the exact herpes zoster incidence occurring in a large sample of patients with HF. The purpose of our study was to investigate whether patients with HF have a higher incidence of herpes zoster than general population. This study provides unique data based on the Longitudinal Health Insurance Database (LHID).

## Methods

### Data source

National Health Insurance (NHI) is a mandatory health insurance program in Taiwan that provides comprehensive coverage for medical care. Up to 99% of the population was enrolled by 2009. All claims data are collected in the NHI Research Database (NHIRD) and are managed by the Taiwan National Health Research Institutes (NHRI).

The NHIRD consists of comprehensive healthcare data provided to researchers, including ambulatory care records, inpatient care records, registration files, catastrophic illness files, and various data regarding drug prescriptions of the insured among 23.5 million Taiwanese residents. Data used to perform the analyses conducted in this study were retrieved from the LHID 2005, a subset of the NHIRD. The LHID2005 consists of all the original medical claims for 1,000,000 enrollees' historical ambulatory data and inpatient care data under the Taiwan NHI program from 1997 to 2010, and the database was created and is publicly released to researchers.

The NHRI, which constructed and maintains the LHID2005 database, further scrambles this encrypted information before the database is released to researchers. The NHRI reported that there were no statistically significant differences in age or gender between the randomly sampled group and all beneficiaries of the NHI program. To maintain claims data accuracy, the NHI’s routine practice of performing cross-checks and validations of medical claims ensures the accuracy of the NHIRD diagnostic coding.

### Study population

We used a study cohort and a comparison cohort to examine the relationship between HF and herpes zoster. We identified 4785 first-time hospitalizations with a discharge diagnosis of HF (International Classification of Disease, 9^th^ Revision, Clinical Modification (ICD-9-CM) codes 398.91, 402.01, 402.11, 402.91, 404.01, 404.03, 404.11, 404.13, 404.91, 404.93, or 428) between January 2001 and December 2009. The date of the initial diagnosis of HF was assigned as the index date for each HF patient. To improve data accuracy, the HF selection criterion required that all case ICD-9 codes by assigned by a cardiologist. We also chose selection criteria for herpes zoster patients. We only included herpes zoster cases in this study if they received ≥ 2 herpes zoster diagnoses for ambulatory care visit or ≥ 1 diagnosis for inpatient care.

Our study used a study cohort and a comparison cohort to examine the relationship between HF and herpes zoster. Each HF cohort patient was matched based on age, gender, and index year to three randomly identified beneficiaries without HF to build the comparison cohort. Patients diagnosed with herpes zoster (ICD-9-CM code 053–053.9) before or after the study period were excluded from both cohorts. We also identified relevant comorbidities, including hypertension (ICD-9-CM 401.X-405.X), diabetes mellitus (ICD-9-CM 250.X), hyperlipidemia (ICD-9-CM 272.X), systemic lupus erythematous (ICD-9-CM 710.0), COPD (ICD-9-CM 491.X), rheumatoid arthritis (ICD-9-CM 714.0), and cancer (ICD-9-CM 140.X-208.X).

### Level of urbanization

For our study of urbanization, all 365 townships in Taiwan were stratified into 7 levels according to the standards established by the Taiwanese NHRI based on a cluster analysis of the 2000 Taiwan census data, with 1 referring to the most urbanized area and 7 referring to the least urbanized. The criteria on which these strata were determined included the population density (persons/km^2^), the number of physicians per 100,000 people, the percentage of people with a college education, the percentage of people over 65 years of age, and the percentage of agricultural workers. Because levels 4, 5, 6, and 7 contained few HF cases, they were combined into a single group, thereafter referred to as level 4.

### Statistical analysis

All data processing and statistical analyses were performed with the Statistical Package for Social Science (SPSS) software, vers. 18.0 (SPSS, Chicago, IL, USA) and SAS vers. 8.2 (SAS System for Windows, SAS Institute, Cary, NC, USA). Pearson *X*^2^ tests were used to compare differences in geographic location, monthly income, and urbanization level of patients’ residences between the study and comparison groups. We also performed a survival analysis using the Kaplan-Meier method, and used the log-rank test to compare survival distributions between cohorts. The survival period was calculated for patients who suffered from HF until an occurrence of hospitalization, an ambulatory visit for herpes zoster, or the end of the study period (December 31, 2010), whichever came first. After adjusting for urbanization level, monthly income, region, and comorbidities as potential confounders, we performed a Cox proportional-hazards analysis stratified by gender, age group, and index year to examine the risk of herpes zoster during the 1-year follow-up in both cohorts. We further classified the gender and age factors in both groups. Hazard ratios (HRs) and 95% confidence intervals (CIs) were calculated to quantify the risk of herpes zoster. The results of comparisons with a two-sided *p* value of < 0.05 were considered to represent statistically significant differences.

### Ethical approval

Insurance reimbursement claims adopted in this study were from Taiwan’s NHIRD, which is available for research purposes. This study was conducted in accordance with the *Helsinki Declaration*. This study was also evaluated and approved by the Kaohsiung Medical University Hospital Institutional Review Board (KMUH-IRB-EXEMPT-20130059).

## Results

**Figure 1:**
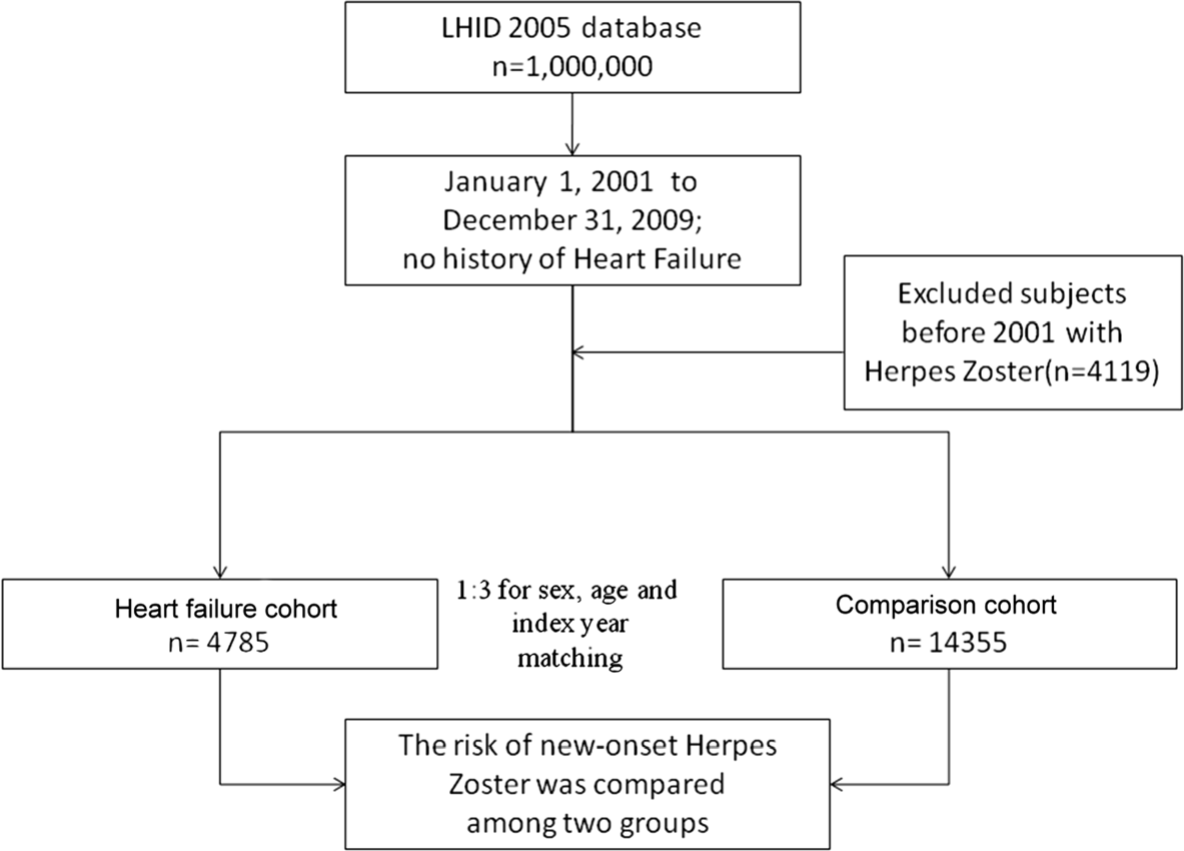
**Flow chart of selection of study subjects and control subjects from the National Health Insurance Research Database in Taiwan.**

The research design of this study is shown in Figure 1. The HF cohort contained 4785 patients, and 14,355 patients were included in the comparison cohort. Distributions of demographic characteristics and comorbidities for the HF and comparison cohorts are shown in Table 1. Hypertension (*p* < 0.001), hyperlipidemia (*p* < 0.001), diabetes (*p* < 0.001), and COPD (*p* < 0.001) were more prevalent in the HF cohort than the comparison cohort. We also found that cases had a greater tendency to have a lower monthly income (*p* < 0.001), and to reside in southern and eastern Taiwan, and reside in less-urbanized communities (*p* < 0.001) compared to controls.

**Table 1:** **Demographic characteristics for the selected patients, stratified by presence/absence of heart failure from 2001 to 2009 (n = 19140)**

	**Patients with heart failure (n = 4785)**		**Patients without heart failure (n = 14355)**		**P value**
	**n**	**%**	**N**	**%**	
**Gender**					1
Male	2547	53.2	7641	53.2	
Female	2238	46.8	6714	46.8	
Age (Years)					1
18-40	197	4.1	591	4.1	
41-65	1359	28.4	4077	28.4	
66-80	2132	44.6	6396	44.5	
81-	1097	22.9	3291	22.9	
**Follow-up, year, mean (SD)**					<0.001
	0.94	0.12	0.996	0.06	
**Urbanization level**					<0.001
1 (most urbanized)	1230	25.7	4396	30.6	
2	1256	26.2	3532	24.6	
3	710	14.8	2164	15.1	
4 (least urbanized)	1589	33.2	4263	29.7	
**Monthly income**					<0.001
0	1572	32.9	4515	31.5	
NT$ 1-15840	1229	25.7	2911	20.3	
NT$ 15841-25000	1695	35.4	5377	37.5	
≧25001	289	6.0	1552	10.8	
**Geographic region**					<0.001
North	1996	41.7	6392	44.5	
Central	1224	25.6	3612	25.2	
South	1191	24.9	3561	24.8	
Eastern	374	7.8	790	5.5	
**Hypertension**					<0.001
Yes	4333	90.6	9606	66.9	
No	452	9.4	4749	33.1	
**Hyperlipidemia**					<0.001
Yes	2472	51.7	6007	41.8	
No	2313	48.3	8348	58.2	
**Diabetes**					<0.001
Yes	2720	56.8	4952	34.5	
No	2065	43.2	9403	65.5	
**SLE**					0.94
Yes	17	0.4	52	0.4	
No	4768	99.6	14303	99.6	
**Rheumatoid arthritis**					0.51
Yes	303	6.3	871	6.1	
No	4482	93.7	13484	93.9	
**COPD**					<0.001
Yes	3265	68.2	6928	48.3	
No	1520	31.8	7427	51.7	
**Cancers**					0.53
Yes	923	19.3	2710	18.9	
No	3862	80.7	11645	81.1	

SD: Standard deviation.

COPD: Chronic obstructive pulmonary disease.

SLE: Systemic Lupus Erythematous.

**Figure 2:**
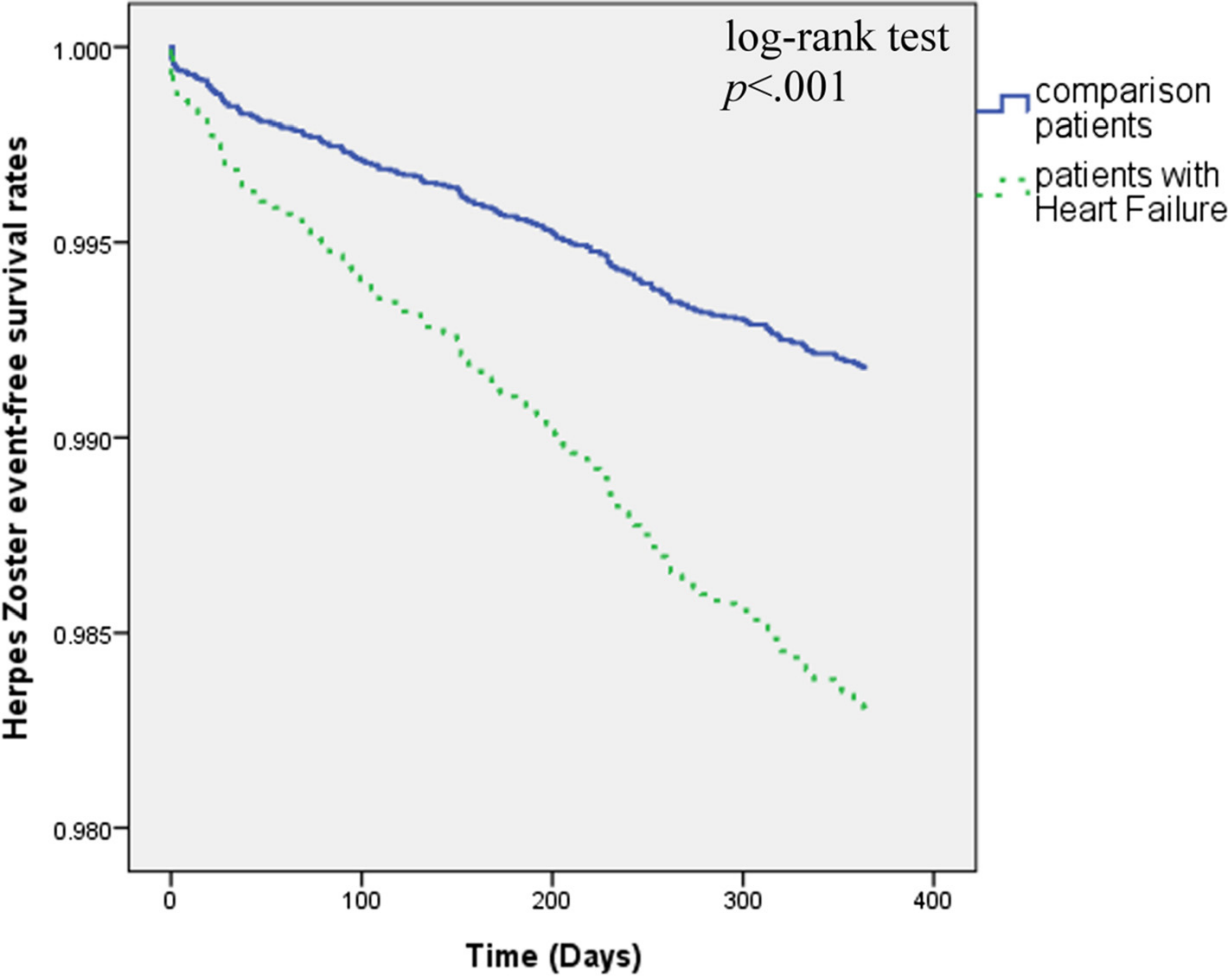
**Herpes Zoster-free survival rates for patient with heart failure patients and comparison groups from 2001 to 2009.**

There were 211 patients diagnosed with herpes zoster during the 1-year follow-up, including 83 HF patients (1.7%) and 128 patients in the comparison cohort (0.9%). The Kaplan-Meier survival curves are shown in Figure 2. The curves demonstrate significantly lower herpes zoster-free survival rates in the HF cohort than in the comparison cohort (log-rank test, *p* < .001). The overall incidence rate was higher in the HF cohort (17.61 per 1000 patient-years) than in the comparison cohort (8.95 per 1000 patient-years).

The Cox regression analysis showed that the crude HR for herpes zoster was 1.96-times greater for HF patients (95% CI: 1.49 ~ 2.59; *p* < 0.001) than for comparison patients. After adjusting for potential confounders, our results showed that newly diagnosed HF was associated with an approximately 2.07-times greater risk of herpes zoster (95% CI: 1.54 ~ 2.78; *p* < 0.001), compared to non-HF patients (Table 2).

**Table 2:** **Hazard ratios (HRs) of Herpes Zoster among heart failure patients during the 1-year follow-up period from the index ambulatory visits or inpatient care from 2001 to 2009**

	**Total**		**Patients with heart failure**		**Patients without heart failure**	
**Development of Herpes Zoster**	**NO.**	**(%)**	**NO.**	**(%)**	**NO.**	**(%)**
1-year follow-up period						
Yes	211	1.1	83	1.7	128	0.9
No	18929	98.9	4702	98.3	14227	99.1
Crude HR (95% CI)				1.96 (1.49-2.59)*	1	
Adjusted HR (95% CI)				2.07 (1.54-2.78)*	1	

Total sample number =19140.

Both crude and adjusted HRs were calculated by Cox proportional hazard regressions.

Adjustments are made for patients’ Sex, Age, Urbanization level, Geographic region, Monthly income, Hypertension, Hyperlipidemia, Diabetes, and Chronic obstructive pulmonary disease.

*Indicates p < 0.001.

We further investigated whether HF is a time-dependent risk factor for herpes zoster and divided HF patients into 3 groups according to the follow-up period. For the three different follow-up periods, there was still statistical significance between the case and comparison groups. The 1-month follow-up period had the highest statistical significance (AHRs: 4.52; 95% CI: 2.28 ~ 8.96; *p* < 0.001) (Table 3). We also further conducted gender- and age-stratified analyses, and results revealed that male subjects (HR: 2.30; 95% CI: 1.51 ~ 3.50; *p* < 0.001) had a higher risk of developing herpes zoster than female subjects (HR: 1.92; 95% CI: 1.27 ~ 2.92; *p* < 0.01). For 41 ~ 65 year olds, there was the highest risk (HR: 3.14; 95% CI: 1.56 ~ 6.21; *p* < 0.01) for developing herpes zoster (Table 4).

**Table 3:** **Hazard ratios (HRs) of Herpes Zoster among heart failure patients during the 1-month, 6-month and 1-year follow-up period from the index ambulatory visits or inpatient care from 2001 to 2009**

	**1-month follow-up period**		**6-month follow-up period**		**1-year follow-up period**	
**Development of Herpes Zoster**	**Patients with heart failure**	**Comparison**	**Patients with heart failure**	**Comparison**	**Patients with heart failure**	**Comparison**
Yes (%)	21 (0.4)	18 (0.1)	50 (1.0)	62 (0.4)	83 (1.7)	128 (0.9)
No (%)	4764 (99.6)	14337 (99.9)	4735 (99.0)	14293 (99.6)	4702 (98.3)	14227 (99.1)
Crude HR (95% CI)	3.51 (1.87-6.59)*	1	2.44 (1.68-3.54)*	1	1.96 (1.49-2.59)***	1
Adjusted HR (95% CI)	4.52 (2.28-8.96)*	1	2.67 (1.79-3.98)*	1	2.07 (1.54-2.78)***	1

Total sample number =19140.

Both crude and adjusted HRs were calculated by Cox proportional hazard regressions, and stratified by age and sex.

Adjustments are made for patients’ Sex, Age, Urbanization level, Geographic region, Monthly income, Hypertension, Hyperlipidemia, Diabetes, and COPD.

*Indicates p < 0.001.

**Table 4:** **Overall and age- and sex-specific incidence rate and relative hazard of Herpes Zoster in the heart failure and comparison cohorts**

**Variable**	**Case group**			**Control group**			**HR**	**AHR**
	**Incident cases**	**Person-year**	**IR** ^**1**^ **per 1000 patient-years**	**Incident cases**	**Person-year**	**IR** ^**1**^ **per 1000 patient-years**		
Age^a^								
18-40	1	196.24	5.10 (−4.87-15.06)	1	590.34	1.69 (−1.62-5.01)	3.00 (0.19-47.92)	7.81 (0.28-215.54)
41-65	23	1341.56	17.14 (10.20-24.09)	22	4066.38	5.41 (3.16-7.66)	3.16 (1.76-5.68)***	3.14 (1.59-6.21)**
66-80	37	2092.79	17.68 (12.03-23.33)	72	6361.81	11.32 (8.72-13.92)	1.56 (1.05-2.32)*	1.66 (1.09-2.53)*
81-	22	1081.41	20.34 (11.93-28.76)	33	3278.74	10.06 (6.65-13.48)	2.02 (1.18-3.46)*	2.26 (1.29-3.96)**
Sex^b^								
Male	42	2514.55	16.70 (11.69-21.71)	61	7612.37	8.01 (6.01-10.02)	2.08 (1.41-3.08)***	2.30 (1.51-3.50)***
Female	41	2197.45	18.66 (13.00-24.32)	67	6684.89	10.02 (7.63-12.41)	1.86 (1.26-2.74)**	1.92 (1.27-2.92)**
Total	83	4712.00	17.61 (13.86-21.37)	128	14297.27	8.95 (7.41-10.50)	1.96 (1.49-2.59)***	2.07 (1.54-2.78)***

IR indicates incidence rate.

^1^Based on the Poisson assumption.

^a^Adjusted for patients’ Sex, Age, Urbanization level, Geographic region, Monthly income, Hypertension, Hyperlipidemia, Diabetes, COPD.

^b^Adjusted for patients’ Age, Urbanization level, Geographic region, Monthly income, Hypertension, Hyperlipidemia, Diabetes, and COPD.

*Indicates p < 0.05; ** Indicates p < 0.01; *** Indicates p < 0.001.

## Discussion

This cohort study demonstrated that, after controlling for other herpes zoster risk factors, patients with HF were at a 2.07 times increased risk than the general population to experience herpes zoster during the 1-year follow-up period. After further stratification by age, gender, and follow-up period, we found a consistent increased herpes zoster risk in HF patients compared to the general population. To our knowledge, this is the first cohort study to investigate the risk of herpes zoster in patients with HF.

Patients with suppressed cell-mediated immunity cause by immunosuppressive disorders or therapies, such as elderly people, patients with autoimmune disease, malignancy, or diabetes, are well described as having a higher risk of developing herpes zoster [14,15,19-24]. Psychosocial stress also appears to reduce immunological control over a latent herpes virus [32], particularly VZV [33]. Patients with advanced HF are associated with many physiological and psychological stressors [34,35], which may increase the risk of herpes zoster. This could partially explain the association between HF patients was increased risk of HZ, especially occurred shortly after the incident HF hospitalization. Besides, natural killer cell dysfunction, which was found in patients with HF [36], was related to reactivation of the HZ in some studies [37]. However, mechanisms that explain the increased risk of herpes zoster in patients with HF remain unclear.

The major morbidity caused by herpes zoster is post-herpetic neuralgia, which consumes a lot of medical resources [38]. The pain of herpes zoster can last for months or years, and treating the complication often requires a multifaceted approach. Besides, several studies document an association between diagnosis of herpes zoster and subsequent diagnosis of cancer [5,6,39,40]. Herpes zoster could be an early manifestation of the immune-system impairment associated with malignancy. It is hypothesis that reactivation of VZV triggers some immunologic mechanisms, such as tissue antigen alteration or antigenic stimulation, which could result in cancer [41]. Determining the risk of cancer following zoster is important because it raises the potential for case finding to facilitate earlier cancer diagnosis. Nowadays, zoster vaccines were documented to markedly reduce the morbidity related to herpes zoster in the immune-competent elderly [42]. As the zoster vaccine was available in Taiwan, the present study provides background data of herpes zoster risk in HF patients, and further studies should focus on the cost-effectiveness of the herpes zoster vaccine in this special population. In addition, we propose a need for further research on the varicella vaccine possibly decreasing relapses of herpes zoster, and associated complications and medical costs in HF patients.

The present study has a number of strengths. First, this is a large-scale follow-up study using the well-established nationwide NHI research database. The study cohort was highly representative of the general population. The medical comorbidities is likely complete and accurate because the NHI is a compulsory and universal healthcare system with a very high coverage rate in Taiwan. In addition, while racial differences are considered to be a factor that influences the risk of herpes zoster [14], approximately 98% of Taiwan’s residents are of Han Chinese ethnicity; this relatively homogenous population reduces potential confounding by race in our study. Furthermore, with the NHIRD, claims for each insured can be tracked across time. In the present study, all claims of different medical institutes during the study period were obtained for analysis. This avoided the bias of patients dropping out that occurs in most longitudinal studies and minimized the possibility of recall bias.

Nevertheless, some insufficiencies should be addressed. First, almost all herpes zoster and HF cases were diagnosed clinically without serologic confirmation or standardized procedure, so diagnoses based on ICD-9-CM codes may be less accurate. However, the NHI Bureau in Taiwan randomly interviews patients and reviews medical records every year to confirm the validity of the diagnoses and quality of care. In addition, the validity of the coding for herpes zoster and HF in administrative data was shown to be good [22,43]. Second, some patients with herpes zoster might have been missed in our database if they did not seek medical help, particularly if their symptoms were mild. Miscoding and misclassification could occur as potential biases. However, utilization of medical services in Taiwan is generally high because there are very low financial barriers to medical care in a very low copayments system. Patients are responsible for a copayment of only US$3 ~ 15 each visit, so most patients with herpes zoster would seek medical attention after disease onset. Thus, the number of herpes zoster cases not included in NHIRD is likely to be small [3]. Third, the claims database lacks information on cigarette smoking, alcohol consumption, dietary habits, physical activities, environmental exposure, nutritional status, and family history, which may confound our findings. In addition, an information bias may arise if the HF patients have a greater tendency to visit physicians than the non-HF patients. Another information bias may occur if physicians are more alert to the diagnosis of herpes zoster in patients with HF than in patients without HF. The administrative database cannot offer information about the HF disease severity, which may also be a confounding factor for the results of the present study. Fourth, the dataset was not directly linked to the national death registry. Therefore, we could not evaluate the mortality between HF and comparison groups during follow-up period. Finally, most residents of Taiwan are of Chinese ethnicity. The ability to generalize our results to other ethnic/racial groups is unclear, and they should be interpreted with caution.

## Conclusions

In conclusion, the present study suggests a positive association between HF and herpes zoster. Physicians, including dermatologists and cardiologists, should be aware of the greater incidence of herpes zoster among patients with HF. They should also be familiar with proper antiviral therapy, as well as acute and chronic pain management. Immune-compromised patients should receive the herpes zoster vaccine for preventive purposes. Future studies are needed to investigate the possible mechanism between different stages of HF and herpes zoster.

## Abbreviations

CI: Confidence interval
HR: Hazard ratio
ICD-9-CM: International Classification of Diseases, Ninth Revision, Clinical Modification
NHI: National Health Insurance
NHIRD: National Health Insurance Research Database
LHID: Longitudinal Health Insurance Database
HF: Heart failure
VZV: Varicella-zoster virus
COPD: Chronic obstructive pulmonary disease
